# The BRAIN Initiative: developing technology to catalyse neuroscience discovery

**DOI:** 10.1098/rstb.2014.0164

**Published:** 2015-05-19

**Authors:** Lyric A. Jorgenson, William T. Newsome, David J. Anderson, Cornelia I. Bargmann, Emery N. Brown, Karl Deisseroth, John P. Donoghue, Kathy L. Hudson, Geoffrey S. F. Ling, Peter R. MacLeish, Eve Marder, Richard A. Normann, Joshua R. Sanes, Mark J. Schnitzer, Terrence J. Sejnowski, David W. Tank, Roger Y. Tsien, Kamil Ugurbil, John C. Wingfield

**Affiliations:** 1 Office of the Director, National Institutes of Health, Bethesda, MD 20892, USA; 2 Howard Hughes Medical Institute and Stanford Neurosciences Institute, Stanford University, Stanford, CA 94305, USA; 3 Howard Hughes Medical Institute and Division of Biology and Biological Engineering, California Institute of Technology, Pasadena, CA 91125, USA; 4 Howard Hughes Medical Institute and Lulu and Anthony Wang Laboratory of Neural Circuits and Behavior, The Rockefeller University, New York, NY 10065, USA; 5 Institute for Medical Engineering and Science and Department of Brain and Cognitive Sciences, Massachusetts Institute of Technology, Cambridge, MA 02139, USA; 6 Department of Anesthesia, Critical Care and Pain Medicine, Massachusetts General Hospital/Harvard Medical School, Boston, MA 02114, USA; 7 Howard Hughes Medical Institute and Department of Bioengineering, Department of Psychiatry and Behavioral Sciences, Stanford University, Stanford, CA 94305, USA; 8 Brown Institute for Brain Science, Brown University, Providence, RI 02912, USA; 9 Biological Technologies Office, Defense Advanced Research Projects Agency, Arlington, VA 22203, USA; 10 Department of Neurobiology, Neuroscience Institute, Morehouse, School of Medicine, Atlanta, GA 30310, USA; 11 Biology Department and Volen Center, Brandeis University, Waltham, MA 02454, USA; 12 Department of Bioengineering, University of Utah, Salt Lake City, UT 84112, USA; 13 Department of Molecular and Cellular Biology, Center for Brain Science, Harvard University, Cambridge, MA 02138, USA; 14 Howard Hughes Medical Institute and James H. Clark Center for Biomedical Engineering & Sciences, CNC Program, Stanford University, Stanford, CA 94305, USA; 15 Howard Hughes Medical Institute and Computational Neurobiology Laboratory, Salk Institute for Biological Studies, La Jolla, CA 92037, USA; 16 Princeton Neuroscience Institute, Bezos Center for Neural Circuit Dynamics and Department of Molecular Biology, Princeton University, Princeton, NJ 08544, USA; 17 Howard Hughes Medical Institute and Department of Pharmacology, University of California San Diego, La Jolla, CA 92093, USA; 18 Center for Magnetic Resonance Research, University of Minnesota, MN 55454, USA; 19 Directorate for Biological Sciences, National Science Foundation, Arlington, VA 22230, USA

**Keywords:** BRAIN Initiative, neural circuitry, neurotechnology

## Abstract

The evolution of the field of neuroscience has been propelled by the advent of novel technological capabilities, and the pace at which these capabilities are being developed has accelerated dramatically in the past decade. Capitalizing on this momentum, the United States launched the Brain Research through Advancing Innovative Neurotechnologies (BRAIN) Initiative to develop and apply new tools and technologies for revolutionizing our understanding of the brain. In this article, we review the scientific vision for this initiative set forth by the National Institutes of Health and discuss its implications for the future of neuroscience research. Particular emphasis is given to its potential impact on the mapping and study of neural circuits, and how this knowledge will transform our understanding of the complexity of the human brain and its diverse array of behaviours, perceptions, thoughts and emotions.

## 1. Introduction

**Figure 1.**
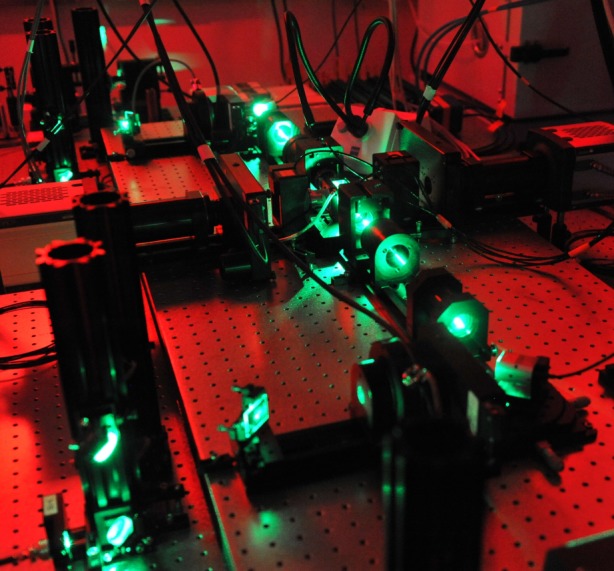
Not Cajal's microscope. Photograph of a current, state of the art, light sheet microscopy system. Image courtesy of Mr Matt Staley and Dr Phillipp Keller, Howard Hughes Medical Institute Janelia Research Campus.

The human brain is the most complex biological entity in the known universe and understanding how it works—that is, how its molecules, cells, circuits and systems enable behaviour, perception, thought and emotion—is the overarching goal of neuroscience. This goal remains elusive, although not from a lack of collective drive or intellectual curiosity on the part of researchers. Rather, progress frequently has been limited by the technologies available during any given era. Over the past decade, however, remarkable technological advances have created entirely new possibilities for studying and understanding the brain. Just as the advent of the microscope enabled Ramón y Cajal to lay the foundation for the ‘neuron doctrine’, innovative technologies from diverse but increasingly convergent disciplines will spur groundbreaking discoveries that will change how we think about the brain (figure 1).

Recognizing that we are on the threshold of a revolution in modern neuroscience, President Obama launched the Brain Research through Advancing Innovative Neurotechnologies (BRAIN) Initiative as a bold new research effort focused on ‘giving scientists the tools they need to get a dynamic picture of the brain in action’ [1]. Given the audacious nature of this goal, the President called for the BRAIN Initiative to be an ‘all hands on deck’ effort involving not only agencies within the United States government, but also companies, health systems, patient advocacy organizations, philanthropists, state governments, research universities, private research institutes, scientific societies and more. The envisioned long-term pay-off of the BRAIN Initiative is a more comprehensive understanding of how the brain produces complex thoughts and behaviours that will provide an essential guide to progress in diagnosing, treating and potentially curing neurological and psychiatric diseases and disorders that devastate so many lives.

As the world's largest funder of biomedical research, it was natural for the National Institutes of Health (NIH) to lead the charge. To ensure a rigorous scientific plan, NIH convened a orking group of neuroscientists—of which we were members—to survey the field and identify key opportunities, milestones and goals for the Initiative's future. While a daunting challenge, members of the working group (hereafter WG) felt privileged to venture outside of our laboratories and clinics, assess the overall state of the field during a period of remarkable change, and make thoughtful recommendations about how to drive the field forward in bold new ways. In this paper, we provide a synopsis of our final report to the NIH, aiming to convey succinctly both the substance and the excitement of our journey. It is particularly appropriate that we share this summary of our scientific vision in a special issue of *Philosophical Transactions* devoted to the future of cerebral cartography.

## 2. The BRAIN Initiative: charting the course

Our charge from the NIH was to ‘catalyse an interdisciplinary effort of unprecedented scope’ that will ‘accelerate the development and application of new technologies to construct a dynamic picture of brain function that integrates neuronal and circuit activity over time and space’ [2]. Throughout our 14-month process, we enriched our discussions and sharpened our insights through broad consultation within and outside the scientific community. We held four summer workshops with invited experts to discuss technologies in chemical and molecular approaches; large-scale recording technologies and structural neurobiology; computation, theory and data analysis; and human neuroscience. Workshop discussions addressed the value of appropriate experimental systems and behavioural analysis—in both animal and human models. Each workshop included opportunity for public comments, which we received both at meetings and on a designated website from multiple sources including patient advocacy groups and members of the general public. The WG issued a preliminary report in September 2013 that recommended high-priority areas for an initial investment of research funding in fiscal year 2014 [3]. The scientific community embraced these findings, and NIH used these initial high-priority areas to craft a set of novel funding announcements focused on developing tools and technologies for visualizing the brain in action, culminating in $46 million of new awards in fiscal year 2014.

The release of the interim report was followed by a period of extended discussion between the WG and the broader community. The leadership of the Society for Neuroscience was consulted and an open town hall held at the Society's annual meeting in November 2013. In addition, we consulted with the presidents of clinical societies in neuroscience-related fields, seeking their advice for the best approaches to the unsolved problems in their fields. The WG met with the leadership of the initial BRAIN Initiative partner organizations, including the NIH, the National Science Foundation, the Defense Advanced Research Projects Agency, the Howard Hughes Medical Institute Janelia Research Campus, the Allen Institute for Brain Science, the Kavli Foundation and the Food and Drug Administration. Open solicitation of advice continued through the NIH BRAIN Initiative website and through numerous one-on-one conversations with colleagues who argued with us throughout the year and educated us in the process. We delivered our final report to NIH in June 2014 [4]. We believe that the salient recommendations and principles articulated in our final report and in the synopsis below represent a ‘best-current-projection’ for a bold and scientifically rigorous BRAIN Initiative.

## 3. BRAIN 2025: a scientific vision

In considering our charge and the current state of neuroscience, the WG identified the analysis of circuits of interacting neurons as being particularly rich in opportunity, with potential for revolutionary advances. This area of research represents a real knowledge gap. We can now study the brain at very high resolution by examining individual genes, molecules, synapses and neurons, or we can study large brain areas at low resolution with whole-brain imaging. The challenge remaining is what lies in between—the thousands and millions of neurons that constitute functional circuits. Analysis of circuits is only one of many areas of neuroscience worthy of attention. However, the WG agreed that accelerating technology development in this area could drive a qualitative shift in what is possible, and progress in this area will benefit many other areas of neuroscience as well. The focus is not on technology *per se*, but on the development and use of tools for acquiring fundamental insight about how the nervous system functions in health and disease. Thus, the WG identified the central challenges of the BRAIN Initiative to be: accelerating the development of technologies for mapping the circuits of the brain, measuring the fluctuating patterns of electrical and chemical activity flowing within those circuits, and understanding how their interplay creates our unique cognitive and behavioural capabilities.

## 4. Priority research areas

The WG identified seven areas of investigation as essential for achieving the ambitious goals of the BRAIN Initiative. In each target area, new technological and conceptual advances are catalysing rapid change in what is achievable, together creating the potential for remarkable new advances. To some extent, segregation into seven target areas is entirely artificial, because each sheds critical light onto the others—a theme that we will bring into sharper focus under #7 (§11). Nevertheless, this division is useful in organizing initial research plans, because many of the immediate goals and experimental techniques are recognizably different from one area to the next.

Throughout our planning process, the WG was acutely aware that the intellectual activity and discoveries that drive the science forward will be made through the initiative of small groups of investigators in hundreds of laboratories around the world, not by a central planning committee. We therefore sought to identify fundamental problems and particularly promising approaches to those problems, not to specify answers or dictate specific actions, which are best left to the initiative of individual research teams. In that spirit, we summarize our seven high-priority target areas and the rationale behind them.

## 5. #1. Discovering diversity: identify and provide experimental access to the different brain cell types to determine their roles in health and disease

The mammalian brain contains approximately 10^8^ (mouse)–10^11^ (human) neurons and even greater numbers of glia [5]. These cells are not homogeneous, but consist of diverse subpopulations with genetically, anatomically, physiologically and connectionally distinct properties [6,7]. Defining these cell types and their functions in different brain regions, and providing methods for experimental access to them in a variety of animals and in humans, is essential to generating a comprehensive understanding of neural circuit function.

A consensus definition and taxonomy of brain cell types has yet to be achieved. Nevertheless, objective classification schemes can be built based on a principled approach combining electrophysiological, gene expression, and anatomical and connectional data [6,8–14]. It is likely that the best working definitions of natural cell types will emerge from empirical classifications based on functionally relevant phenotypic dimensions [7,14,15]. Working definitions will be updated continuously as more data are collected and a deeper understanding emerges.

Important contributions to the conceptual definition of cell type will also come from iterative interactions with theory and modelling. For example, theoretical considerations may specify the level of granularity of cell type identification that is necessary to understand the computations in a particular brain region [16], providing a guide for experimental analysis. In turn, the level of cellular heterogeneity observed in a given brain region can constrain models and generate new predictions concerning circuit function or disease intervention.

More concretely, we believe that a cell types inventory should focus initially on a few key brain regions in model organisms such as *Caenorhabditis elegans*, *Drosophila*, zebrafish, mouse and non-human primate. The inventory should include molecular, anatomical and electrophysiological descriptions as well as the development of tools for genetic access to all of these cells. The model organisms and brain regions should be prioritized based on their interest to large communities of neuroscience researchers and their relevance to behaviour and human disease. In the mouse, for example, relevant areas might include the retina, spinal cord, hippocampus, striatum, amygdala/hypothalamus and prefrontal cortex. This strategy would identify challenges and opportunities for iterative tactical improvements in technology and process. A long-term goal of this project—on the order of 10 years out—is to achieve proof-of-principle cell type-specific targeting for therapeutic manipulations in humans.

A comprehensive, rigorous inventory of cell types will enable neuroscientists to begin answering questions of fundamental importance such as

— What level of granularity of cell type definition is necessary for understanding the function of a given neural circuit?
— What are the fundamental principles guiding the organization of the various cell types throughout the brain?
— Do well-defined cell types shape neural circuit function to a greater extent in some brain regions than in others?
— Can we target specific human cell types to develop new therapies for neurological and psychiatric disorders?

### (a) Summary

It is within reach to characterize all cell types in the nervous system, and to develop tools to record, mark and manipulate these precisely defined neurons in the living brain*.* We envision an integrated, systematic census of neuronal and glial cell types, and new genetic and non-genetic tools to deliver genes, proteins and chemicals to cells of interest in non-human animals and in humans. Many substantive problems must be solved to achieve this goal, including increasing the throughput, scale and dimensionality of cellular phenotyping; increasing the specificity of experimental access; creating new abilities to measure the stability of molecular properties across time scales and dimensions and the standardization of methods across laboratories. These challenges notwithstanding, the general path forward is clear.

## 6. #2. Maps at multiple scales: generate circuit diagrams that vary in resolution from synapses to the whole brain

Throughout the brain, the flow and processing of information is mediated by anatomical connections that unify cells into circuits and circuits into systems. These connections include both local connections within a specific brain region and long-range connections spanning multiple areas and distances. Maps of anatomical connectivity at various levels of detail provide an essential foundation for understanding the functional signals underlying cognition and behaviour [17].

Existing methods for mapping anatomical connectivity have provided numerous valuable insights but remain inadequate for making the next leap in understanding. For instance, our direct knowledge of human brain connectivity derives almost exclusively from macro-connectomic measurements based on magnetic resonance imaging, but these methods achieve at best a spatial resolution of 2 mm (isotropic), provide only indirect measures of connectivity, and yield little information about directionality [18–20]. Improvements in resolution of macro-connectomic techniques are feasible and would enable new sets of questions to be asked about human brain connectivity. In addition to improved resolution, another high priority for the near future is to validate macro-connectomic maps in animal models where macro-scale maps can be compared directly to finer scale maps.

**Figure 2.**
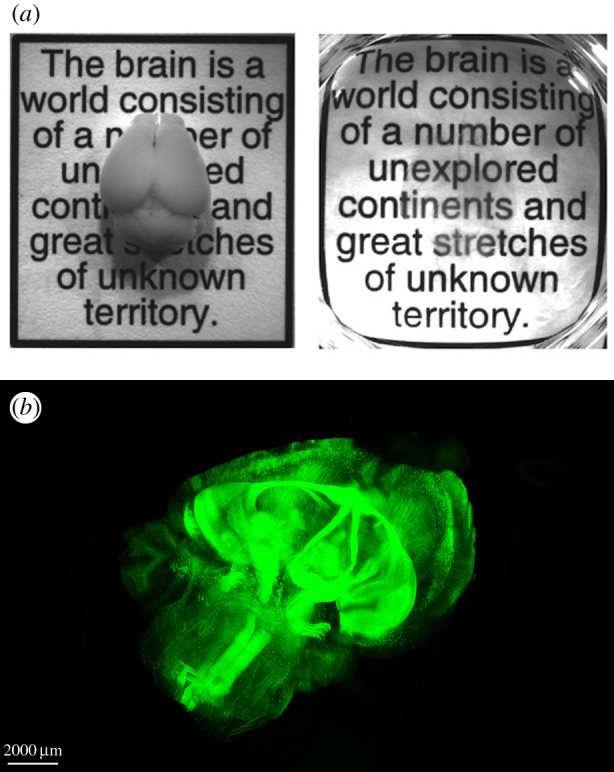
Integrating neuroscience and chemical engineering—CLARITY technique. Images courtesy of the Deisseroth Laboratory, Stanford University. (*a*) Mouse brain prior to CLARITY transformation (left). Following CLARITY, the brain is rendered transparent while preserving its structural integrity (right). (*b*) High-resolution fluorescence signals (as well as antibody labels and oligonucleotide probes) pass entirely through intact brains in CLARITY (shown is an adult mouse brain with long-range projections labelled in green with a genetic marker).

Meso-connectomic approaches (millimetre-to-micrometre resolution), capable of mapping both local and interarea connections with cellular resolution, provide the bulk of our knowledge of anatomical connectivity in animal models. Ongoing efforts to apply these techniques systematically to large numbers of brain structures, and with cell-type specificity, are extremely valuable [21,22]. Retrograde and anterograde trans-synaptic tracers are beginning to allow direct study of cell-to-cell connectivity at the mesoscale, which is essential for identifying repeating circuit motifs that characterize individual brain structures or are common across brain structures [23]. However, these methods are inadequately validated and viral vectors are toxic to living tissue. The greatest need in this area is to improve the specificity and reliability of methods for projectome and cell-type-to-cell-type tracing. Newer methods that render the brain transparent and leave its connections intact, such as CLARITY [24], Scale [25], SeeDB [26] and others [27–29], are poised to make a significant impact (figure 2). Importantly, mesoscale connectivity maps have the greatest potential for direct alignment with large-scale activity measurements obtained through calcium imaging [30]. To make this vision a reality, we will need new optical and computational methods for efficient, high-resolution collection of multidimensional anatomical datasets from large brain volumes and registration of these datasets with cellular-resolution activity information (see #7).

Micro-connectomic maps (micrometre-to-nanometre resolution), involving dense electron microscope reconstruction at level of individual cells and synapses, are considered by many neuroscientists to be the gold standard for connectomics. Currently, it takes orders of magnitude more time to generate electron microscope reconstructions than it does to obtain the original data, resulting in data *analysis* rather than data *acquisition* being the most significant barrier to progress [11,31–33]. The BRAIN Initiative must spur improvements in segmentation and reconstruction methods; machine learning, crowd-sourcing and other promising approaches can be pursued in parallel to accelerate the image analysis process. Importantly, segmentation methods developed in this effort will be applicable to light as well as electron microscopic datasets.

The first few years of the BRAIN Initiative will focus on improving current technologies at all scales and exploring the broadest possible space in search of new technologies. In later years, we anticipate that these improved technologies will enable complete sparse reconstructions of key areas in brains of normal animals and in selected animal models of human brain disorders. Likewise, we anticipate that initial segmentation of key areas within typical and pathological human brains will be feasible within 10 years. Combining these datasets into a common bioinformatic framework and registering them with other streams of information describing the cell populations and projections of interest, such as molecular phenotype and activity patterns during behaviour, will increase their depth and scientific utility.

Armed with improved maps at all three scales we can begin to answer the following questions

— Can macro-connectomic connectivity patterns serve as biomarkers for human brain disorders? How widely can they be used for differential diagnosis of brain disorders, monitoring disease progression, and predicting or monitoring responses to therapy?
— Do some brain disorders result from anatomical ‘connectopathies’ with stereotyped defects in neural circuitry?
— What changes in circuits accompany, and perhaps cause, age-related cognitive decline?
— How different are connectivity patterns in genetically identical organisms in a species, ranging from isogenic flies and worms to identical twin humans?
— Can individual variations in connectivity be related to or even predict individual differences in brain function?

### (a) Summary

It is increasingly possible to map connected neurons in local circuits and distributed brain systems, enabling an understanding of the relationship between neuronal structure and function. We envision improved technologies—faster, less expensive, scalable—for anatomic reconstruction of neural circuits at all scales, from non-invasive whole human brain imaging to dense reconstruction of synaptic inputs and outputs at the subcellular level.

## 7. #3. The brain in action: produce a dynamic picture of the functioning brain by developing and applying improved methods for large-scale monitoring of neural activity

Large-scale monitoring of neural activity is fundamental to the goals of the BRAIN Initiative, providing critical data for measuring and understanding the changes in electrical and chemical signalling underlying mental processes and behaviour. Ultimately, neural population activity must be measured across dispersed circuits, diverse cell types and at multiple timescales, creating unique technological challenges.

Currently, there are two important classes of methods for recording neuronal activity with cellular resolution, both possessing their own limitations. Classically, direct measurement of electrical activity with microelectrodes has been the workhorse tool. We are still far from the goal of dense recordings from highly complex circuits, even though microelectrode recording methods have been scaled up in recent years [34–37]. For example, large-scale neurophysiological approaches have allowed recordings from thousands of neurons in the vertebrate retina, but even so only the retinal ganglion cells have been recorded at scale, not the many non-spiking bipolar and amacrine cells that process information prior to optic nerve output [38]. More recently, optical sensors (e.g. chemical, genetic) for recording activity have been greatly improved and have proved to be a tremendous asset for monitoring activity in large numbers of densely packed neurons [39–42]. Optical tools capture a central vision of the BRAIN Initiative, in that they may ultimately facilitate the integration of many multiple approaches into a single experiment—activity monitoring, activity manipulation, circuit reconstruction and characterization of a single cell's morphology and molecular constituents (or at least a subset of the above). Optical methods also have the greatest potential for measuring chemical and biochemical processes in the brain, an important complement to measurements of electrical activity.

Both electrical and optical methods will continue to be essential in studying neural circuitry and we urge a continued focus on improving these techniques. New and improved electrode arrays should increase the number and density of recorded neurons, provide access to more brain areas, increase reliability and improve biocompatibility through better materials and electrode design. Great benefit could come from short-term efforts to improve optical sensors to enhance the speed, tissue volume, tissue depth [43] and the number of brain regions that can be monitored in living animals. Ultimately, new recording technologies will provide the ability to map at unprecedented resolution and scale the electrical and chemical activity of populations of neurons in the *awake brain* during cognition, emotion and behaviour. These new data will provide the basis for a conceptual understanding of neural coding: how information relevant to the brain state, sensory stimuli or other variables are encoded in neural population activity. Following the changes in neural activity over time—circuit dynamics—will provide key information for establishing the computational function of a neural circuit, and allow us to develop and test (when combined with causal manipulations) hypotheses about the necessity and sufficiency of particular neural activity patterns for specific behaviours and cognitive processes.

In the intermediate or long-term, revolutionary new recording technologies may emerge, and we encourage their exploration and development. Future development of next-generation recording technologies will increasingly require the participation of scientists from physics, chemistry, molecular biology, electrical and neuroengineering, materials science and computer science. Development of a new generation of large-scale recording tools will permit more incisive investigation of numerous problems in neuroscience that have been approached in only limited ways to date

— How is sensory information transformed into higher-order perception?
— How is short-term working memory encoded, maintained, and read out?
— What are the circuit mechanisms underlying decision-making?
— What fundamental logic and neural mechanisms mediate motor control?
— How do multiple brain areas communicate and work together as behaviour and task demands change?
— How can we reliably detect internal brain states that are not time-locked to externally observable events? What are the unique functions of these states?
— How do neuromodulatory signals remodel circuit dynamics and brain states?
— How are internal cognitive models of the world encoded, updated, and accessed to make predictions and guide future actions?

### (a) Summary

We should seize the challenge of recording dynamic neuronal activity from densely sampled—and in some test cases complete—neural networks, over long periods of time, in all areas of the brain, in both mammalian systems and diverse model organisms. There are promising opportunities both for improving existing technologies and for developing entirely new technologies for neuronal recording, including methods based on electrodes, optics, molecular genetics and nanoscience, and encompassing different facets of brain activity.

## 8. #4. Demonstrating causality: link brain activity to behaviour by developing and applying precise interventional tools that change neural circuit dynamics

Observing natural patterns of neural activity during behaviour generates hypotheses about their functional significance, but causal tests of such hypotheses require direct manipulation of the underlying activity patterns. Precise circuit-level perturbation techniques are needed to (i) determine the causal significance of neural activity patterns in cognition, emotion, perception and other processes; (ii) probe the internal structure and dynamics of nervous systems; and (iii) serve as a basis for new therapeutic interventions.

A major recent advance in circuit manipulation has been the development of optogenetic tools based on light-activated channels and ion pumps [44,45]. The combination of rapid activation, reliable effects and genetic delivery of the light-sensitive channels to specific cell types and brain regions has proved to be a general method for testing and generating hypotheses of brain function across systems, brain regions and (non-human) species. There are broader possibilities for manipulating neuronal activity *in vivo.* Chemogenetic tools (such as Receptors Activated Solely by a Synthetic Ligand (RASSLs), Designer Receptors Exclusively Activated by Designer Drugs (DREADDs) and chemical–genetic switches for kinases and channels) [46] are already a useful complement to optogenetics and thermally regulated tools [47] for long-term manipulation, and this is another area that will benefit from continued improvement [46].

New and improved perturbation tools could rapidly amplify research efforts around the world. Optogenetics, for example, is limited by light scattering, typically requiring fibre optics for most deep brain structures [44]. Potential improvements include narrower (light wavelength) action spectra, increased light-sensitivity and tools with new kinds of ion conduction properties or other electrical or biochemical modulatory capabilities. Optogenetic approaches also need to develop further to enable not just cell-type resolution, but single-cell resolution, in systems as complex as behaving animals [48–50]. Similarly, new chemogenetic tools with an expanded repertoire of effectors and more time-resolved kinetic properties would significantly advance our capabilities for targeted circuit perturbation, and could open the door to additional approaches for understanding and treating brain disease [51,52].

Invention of completely novel perturbation approaches is badly needed, especially for non- (or minimally) invasive use in the human nervous system. Novel tools might be based on magnetic stimulation, gases, infrared excitation, ultrasound, synthetic biology or organic or physical chemistry to allow access to neurons deep within the brain. Again, future efforts should increasingly emphasize integration of major technical approaches—cell type access, connectomics, recording and perturbation—into seamless investigation of fundamental questions about neural circuit function.

Finally, the analysis of behaviour must improve in its spatial and temporal precision to match the precision of activity measurements and manipulations. Innovations in psychophysics, machine learning and virtual reality should be encouraged to create a rich understanding of the brain's output in behaviour.

Novel perturbation tools developed over the past decade have had a remarkable impact on experimental neuroscience. New and improved tools will continue to re-make the field, offering neuroscientists the opportunity to address fundamental questions from novel points of view

— How are measureable aspects of perception and behaviour modulated by alteration of activity patterns in underlying neural populations?
— What alterations of these activity patterns give rise to maladaptive or pathological behaviour?
— Are precise corrections of these activity patterns at the cellular level necessary to restore typical behaviour, or are more simple shifts in regional or projection dynamics sufficient?
— What is the causal role of spike rate, timing and synchrony relationships among neurons, projections and brain regions, in circuit processing and behaviour?
— Are there consistent neural activity ‘motifs' or patterns that perform core computations in different brain regions that perform different tasks?
— Can therapeutic intervention be productively guided in a patient-specific way by considering brain structure or activity alongside a patient's symptoms, and then adapting an activity intervention to that patient's unique clinical situation?

### (a) Summary

By directly activating and inhibiting populations of neurons in a behavioural context, neuroscience is progressing from observation to causation, and much more is possible. To enable the immense potential of circuit manipulation, a new generation of tools for optogenetics, chemogenetics and biochemical and electromagnetic modulation should be developed for use in animals and eventually in human patients.

## 9. #5. Identifying fundamental principles: produce conceptual foundations for understanding the biological basis of mental processes through development of new theoretical and data analysis tools

The overarching goal of theory, modelling and statistics in neuroscience is to create an understanding of how the brain works—how information is encoded and processed by the dynamic activity of specific neural circuits, and how neural coding and processing lead to perception, emotion, cognition and behaviour. Powerful new experimental technologies developed in response to the BRAIN Initiative will produce datasets of unprecedented size and sophistication, but rigorous statistical analysis and theoretical insight will be essential for understanding what these data mean. Coherent lessons must be drawn not only from the analysis of single experiments, but also by integrating insights across experiments, scales and systems. Theoretical studies will allow us to check the rigour and robustness of new conceptualizations and to identify distinctive predictions of competing ideas to help direct further experiments. Neuroscience will mature to the extent that we discover basic principles of neural coding and computation that connect and predict the results of multi-modal experimental manipulations of brain and behaviour.

To extract meaning from large datasets efficiently, new techniques for analysing large, complex datasets need to be developed, including methods for finding high-order structure in recording, anatomical and behavioural datasets; methods for building models that can identify potential underlying mechanisms; and methods for rigorous hypothesis testing and inference by fitting models to data [53–57]. These new analytic and modelling techniques are likely to prove beneficial in every stage of research under the BRAIN Initiative, from experimental design to final analysis and interpretation, so that expensive, multidimensional datasets can optimally address the questions being asked. Ultimately, sophisticated statistical and computational techniques need to be made available to all neuroscientists at all levels, faculty, postdoctoral and graduate student, to generate increased quantitative rigour throughout the field.

**Figure 3.**
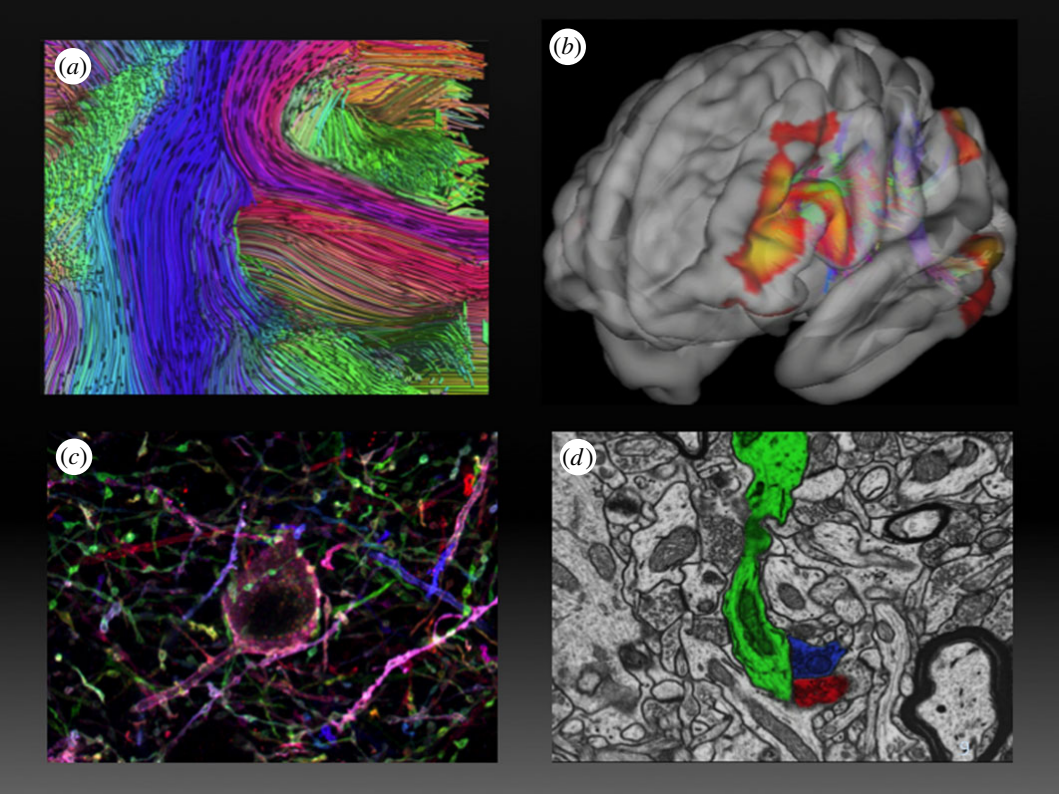
Spatial scales of structural analysis. (*a*) Macro-connectomics. Diffusion-weighted magnetic resonance imaging (DW MRI) with approximately millimetre spatial resolution (voxel volume 1 × 1 × 1 mm cubed) enables non-invasive mapping of long distance tracts within the entire human brain, which can then be related to functionally defined regions in functional magnetic resonance imaging (fMRI) experiments in the same spatial scale, as in (*b*). (*c*) Meso-connectomic approaches are capable of mapping both local and interarea connectivity at cellular resolution (micrometre spatial scale). (*d*) Dense electron microscopic reconstruction with nanometre in-plane resolution and serial slices of 50–100 nm enables micro-connectomic mapping of circuitry at the level of individual cells and synapses. Relating these three levels of structural analysis to each other and to data streams from genetic, electrophysiological, optical, perturbation, behavioural, etc. experiments is a central challenge of the BRAIN Initiative. (*a*, *b* and *d*) Courtesy of Dr Kamil Ugurbil, University of Minnesota ((*a*) from [59], (*b*) generated from the Washinton University, University of Minnesota Human Connectome Project data by Saad Jbabdi, Oxford University; (*d*) from supplemental data supplied in [60]). (*c*) Courtesy of Dr Joshua Sanes, Harvard University, and Dr Dawen Cai, University of Michigan.

A particularly daunting challenge for the BRAIN Initiative is to integrate data across multiple scales of space and time [58]. Datasets from different laboratories will cover spatial scales ranging from micrometres to metres (figure 3), and time scales ranging from milliseconds to minutes, hours or even the lifetime of an organism. New analytic and computational methods, as well as new theoretical frameworks, are fundamental in understanding how organism-level cognition and behaviour emerge from the interplay of structural connectivity and signalling events at the molecular, cellular and circuit levels. By synthesizing results from numerous experiments that explore neural circuits at different levels, theoretical studies should uncover common themes and general principles. These principles will elucidate how neural circuits work, that is, how populations of neurons collectively support the brain's many functions.

Among the key questions to be addressed by a unified theoretical and experimental approach under the BRAIN Initiative are

— What are the neural codes used by brains for sensory information processing, information transmission and motor control?
— How do interacting neurons in distributed circuits integrate and transform inputs on multiple time scales?
— How are neural dynamics changed by learning? What are the modulatory and plasticity mechanisms responsible for different forms of learning?
— What neuronal, synaptic, biochemical and circuit mechanisms support working memory and long-term memory? How are memories retrieved?
— How do neural circuits ‘read out’ the dynamics of multiple neural populations to guide behaviour and cognition?

### (a) Summary

Rigorous theory, modelling and statistics are advancing our understanding of complex, nonlinear brain functions where human intuition fails. New kinds of data are accruing at increasing rates, mandating new methods of data analysis and interpretation. To enable progress in theory and data analysis, the BRAIN Initiative must foster collaborations between experimentalists and researchers from statistics, physics, mathematics, engineering and computer science.

## 10. #6. Advancing human neuroscience: develop innovative technologies to understand the human brain and treat its disorders; create and support integrated human brain research networks

Each goal of the BRAIN Initiative has an explicit component addressing human brain research and, accordingly, sections of our report addressed technologies to study human cell types, connectivity, large-scale activity, functional perturbation and models of brain function. Beyond these topics, however, there are scientific, experimental and ethical issues that are specific to human neuroscience, whether fundamental, translational or clinical.

Studies of human brain activity present extraordinary opportunities for both clinical advances and scientific inquiry. For example, clinically approved investigational technologies, including devices that are surgically implanted into the brain, provide a unique opportunity to investigate neural function by recording and/or stimulating at the resolution of cells and circuits. Implanted devices are being used in clinical practice to monitor brain function, to diagnose and treat mood and movement disorders, and to restore sensory and motor functions lost following injury or disease [61–65]. When coupled with non-invasive measurements of functional activity—via MRI, electroencephalography, magnetoencephalography etc., there are real opportunities to bridge scales from (limited) cellular to whole brain functional imaging methods. However, much of this exceptionally valuable human data is not captured, curated or made available for research. The important possibility of carrying out research on human brain function while advancing the clinical capabilities of emerging neurotechnologies creates special issues for human research, including

— *Clinical support networks*. A means to ensure support of fundamental human brain research in clinical settings and within clinical trials.
— *Training.* Understanding of the special requirements for human research. Training a new generation of neuroscientists who are rigorous researchers, compassionate clinicians, creative engineers and adept administrators of complex scientific teams.
— *Data capture and sharing.* A means to capture human data in standardized formats and to curate and share these data within the framework of protecting private information.
— *Effective human neurotechnology.* A means to advance the development of safe, but innovative technology suitable for research in human brains.
— *Ethics.* Strong ethical frameworks, review and oversight of human research.

Integrated research teams of clinicians, scientists, device engineers, patient-care specialists, regulatory specialists and ethics specialists are needed to capitalize fully on the unique research opportunities offered by studies in which informed, consenting human subjects participate. Such teams may be assembled within a single university or medical centre, or may comprise integrated consortia across multiple universities and medical centres, which could facilitate sharing of standardized data and training in this research. Industry input and collaboration are also highly valued; this sector often possesses specialized expertise in overcoming hurdles to effective translation.

We believe that newer iterations of technology development should focus on maximizing scientific research value while advancing clinical diagnostics or therapeutic applications. The most dramatic improvement that could be made for implanted devices would be to combine multiple measurements and manipulation capabilities in a single device (e.g. combined recording and stimulation capabilities). Next-generation devices should be inspired by recent advances in electrical, optical, acoustic, genetic and other modalities.

Some of the unique questions that could be addressed by human neuroscience research include

— How does neural activity in specific circuits relate to the conscious experience of humans as they perform a cognitive or behavioural task?
— What neural mechanisms underlie the remarkable human ability to represent information symbolically (as in language) and then use that information in novel situations outside the context in which it was originally learned?
— What patterns of neural activity in which brain structures correspond to human emotional states? Can we treat emotional disorders by applying neuromodulation techniques to these structures and circuits?
— Can we decode mental motor plans with sufficient speed and accuracy to control supple, effective prosthetic devices for paralysed patients?

### (a) Summary

Consenting humans who are undergoing diagnostic brain monitoring or receiving neurotechnology for clinical applications provide an extraordinary opportunity for scientific research. This setting enables research on human brain function, the mechanisms of human brain disorders, the effect of therapy and the value of diagnostics. Seizing this opportunity requires closely integrated research teams performing according to the highest ethical standards of clinical care and research. New mechanisms are needed to maximize the collection of this priceless information and ensure that it benefits both patients and science.

## 11. #7. From BRAIN Initiative to the brain: integrate new technological and conceptual approaches produced in goals #1–6 to discover how dynamic patterns of neural activity are transformed into cognition, emotion, perception and action in health and disease

The overarching vision of the BRAIN Initiative is best captured by this priority research area—combining multiple approaches into a single, integrated science of cells, circuits, brain and behaviour. In some cases, particularly, in the early phases of the BRAIN Initiative, a given technology will be applied in isolation to provide important new knowledge. However, many of the most exciting and powerful applications will come from combining the new technologies as parts of an integrated whole, a goal we have pointed to in several preceding sections. For example, immense value is added if recordings are conducted from identified cell types whose anatomical connections are established in the same study. Such an experiment is currently an exceptional tour de force; with new technology, it could become routine. In another example, neuronal populations recorded during complex behaviour might be immediately retested with circuit manipulation techniques to determine their causal role in generating the behaviour. Hand-in-hand with these new combinations of experimental methods will come integrated work from theory, modelling and statistics that provide rigour to observations, new methods of visualization and understanding of the data and, most importantly, new conceptual frameworks for interpretation of the data. When verified, these hypotheses and theories will provide the mechanistic understanding of neural circuit function that is the principal goal of the BRAIN Initiative.

Integration of neuroscience technologies in the BRAIN Initiative should emphasize a systems engineering approach, in which one optimizes the collective performance of the final system, rather than optimizing individual components [66]. For example, a systems engineering approach to developing fluorescent indicators of neural activity should focus not only on the properties of the indicator molecule, but also on parameters such as wavelength compatibility with other optical sensors, optogenetic probes, detectors or illumination sources, or robustness to fixation processes used in post mortem analyses. This would be important when combining targeted recording with *post hoc* connectomic analysis. Similarly, designers of new lasers, lenses or detectors for deep tissue imaging should consider the capabilities of important molecular sensors and probes.

### (a) Summary

The most important outcome of the BRAIN Initiative will be a comprehensive, mechanistic understanding of brain function that emerges from synergistic application of the new technologies and conceptual structures developed under the BRAIN Initiative.

## 12. Core principles of the BRAIN Initiative

Over the course of our deliberations, specific themes emerged regarding core principles for the BRAIN Initiative. We believe that these principles are integral to more rapid, effective advances in understanding the brain, ultimately leading to treating neurological and neuropsychiatric disorders. Some of these principles have been articulated already by other international and private brain research initiatives. Although formulated in a specifically American context, we suggest that these general principles might also be suitable for guiding other brain research efforts.

(1) *Pursue human studies and non-human models in parallel.* The goal is to understand the human brain, but many methods and ideas are developed first in animal models, both vertebrate and invertebrate. Experiments should take advantage of the unique strengths of diverse species and experimental systems.
(2) *Cross boundaries in interdisciplinary collaborations.* No single researcher or discovery will solve the brain's mysteries. The most exciting approaches will bridge fields, linking experiment to theory, biology to engineering, tool development to experimental application, human neuroscience to non-human models in innovative ways.
(3) *Integrate spatial and temporal scales.* A unified view of the brain will cross spatial and temporal levels, recognizing that the nervous system consists of interacting molecules, cells and circuits across the entire body, and important functions can occur in milliseconds or minutes, or take a lifetime.
(4) *Establish platforms for preserving and sharing data.* Public, integrated repositories for datasets and data analysis tools, with an emphasis on ready accessibility and effective central maintenance, will have immense value.
(5) *Validate and disseminate technology.* New methods should be critically tested through iterative interaction between tool-makers and experimentalists. After validation, mechanisms must be developed to make new tools available to all.
(6) *Consider ethical implications of neuroscience research.* BRAIN Initiative research may raise important issues about neural enhancement, data privacy and appropriate use of brain data in law, education and business. These important issues must be considered in a serious and sustained manner. BRAIN Initiative research should adhere to the highest ethical standards for research with human subjects and with non-human animals under applicable federal and local laws.
(7) *Create mechanisms to ensure accountability to the government sponsor* (*NIH in our case*), *the taxpayer and the basic, translational and clinical neuroscience communities.* The BRAIN Initiative is extremely broad in interdisciplinary scope and will involve multiple partners. Oversight mechanisms should be established to ensure that BRAIN funds are invested wisely for the ultimate benefit of the public and the scientific community.

## 13. Concluding remarks

We are at a unique moment in the history of neuroscience—a moment when technological innovation has created possibilities for discoveries that could cumulatively lead to a revolution in our understanding of the brain. For some of our goals, novel technologies are already in place and simply need to be exploited at scale and in a highly coordinated fashion. In other cases, however, entirely new technologies need to be envisioned and created, especially for non-invasive, high-resolution recording and modulation of human brain circuits. We believe that these goals are achievable with sufficient investment of human and financial resources and scientific infrastructure, all of which are critical to the success of the BRAIN Initiative. An investment that builds up over 5–7 years and is sustained through time will encourage talented scientists to form new collaborations to solve important, difficult problems because they will perceive a long-term commitment of the NIH and other US government agencies.

The US commitment will be markedly enhanced by the rise in complementary efforts around the globe, such as the European Union's Human Brain Project, Japan's Brain/MINDS (Brain Mapping by Integrated Neurotechnologies for Disease Studies) project, and CanadaBrain—to name just a few. Planning is also underway for a national brain project in China. It will be important for the research community to continue to engage in a regular dialog about the scientific opportunities and challenges associated with these large-scale efforts. Regular communication will be key for researchers to learn from each other's success and failures, and identify potentially synergistic research approaches when appropriate.

Like the Apollo program, undertaking a grand challenge of this sort will require the development and integration of an array of new technologies, drawing on scientists and engineers from a multitude of disciplines. The neuroscience ‘moonshot’ differs, however, in that we cannot foresee exactly where these new technologies and experiments will take us. Charting a course to the moon was far simpler than unlocking the mysteries of our own minds! Future technological innovation will certainly propel neuroscience research in entirely new, unexpected directions, as it has so often in the past. Consider, for instance, the evolution of the field of cerebral cartography, the focus of this special issue. The first era of systematic brain mapping, begun by Fritsch and Hitzig in the 1870s [67], culminated in Wilder Penfield's systematic electrical stimulation studies in conscious human patients undergoing surgery for epilepsy [68]. These early experiments demonstrated beyond all doubt the localization of function within the cerebral cortex, including the primary sensory and motor areas, specialized language areas, hemispheric laterality and neural systems involved in memory. More penetrating studies awaited the invention of the intracortical microelectrode and anatomical tracer techniques based on anterograde and retrograde axoplasmic transport. With the advent of these tools, neuroscientists were able to explore the exquisitely selective response properties of single neurons [69,70], identify the cortical column as a primary unit of cerebral information processing [69,71] and discover a host of new cortical areas and the anatomical circuits that connect them [72–75]. The development of techniques for recording from awake, behaving animals [76] and for non-invasive imaging of the human brain [77–79] and animal brains [80] led to the rise of cognitive neuroscience in the last quarter of the twentieth century, bringing previously mysterious cognitive process such as attention, working memory, spatial navigation, decision-making and motor planning under direct empirical examination. The fusion of molecular and cell biological techniques with *in vitro* slice recordings enabled elucidation of many basic mechanisms of synaptic plasticity.

As exhilarating at these discoveries have been, the best is certainly yet to come. The pace of technological change in neuroscience has accelerated dramatically in the past decade, giving researchers powerful new tools to access and manipulate specific types of cells, analyse coding and circuit dynamics across extended neural populations, and causally link specific patterns of neural activity to cognition and behaviour. The sophisticated new datasets and circuit manipulations emerging from neuroscience laboratories around the world—nearly unimaginable a decade ago—are providing rich fodder for theorists and modellers seeking to identify the biophysical and circuit-level principles underlying nervous system function. The BRAIN Initiative will accelerate this cycle of technical innovation and scientific discovery by fostering integration of the new experimental approaches with each other and with theory and modelling, creating a path towards solving the mysteries of the brain's circuits and their activity across time and space.

Understanding the brain is a worthy goal in and of itself. But, in the longer term, new treatments for devastating brain diseases are likely to emerge from a deeper understanding of the brain. For example, treatment of Parkinson's disease has been greatly enhanced by circuit-level understanding of the brain's motor systems. Our front-line treatment for Parkinson's is the dopamine precursor drug, l-DOPA, but its efficacy decreases over time while severe side effects increase [81]. In response, teams of neurophysiologists, engineers and physicians fused an understanding of the brain's motor circuits with technological advances to create deep brain stimulation, which can restore motor circuit function in many Parkinson's patients for up to several years [82]. Current research into brain circuits for mood and emotion has the potential to advance psychiatry in similar ways [63].

Powered by novel technological capabilities, neuroscience has gathered remarkable momentum over the past decade. We believe that great leaps forward can be made in just a few years or decades given an infusion of new interdisciplinary talent, coordination of effort, and investment of resources at the national level. Like other great leaps in the history of science—the development of atomic and nuclear physics, the elucidation of the genetic code—this one will change human society forever. Through deepened knowledge of how our brains actually work, we will understand ourselves differently, treat disease more incisively, educate our children more effectively, practice law and governance with greater insight and develop more understanding of others whose brains have been moulded in different circumstances. To achieve this vision, we must train and support a new generation of cross-disciplinary brain scientists and provide the resources needed to unleash their creative energies for the benefit of all.
